# Summary of the National Advisory Committee on Immunization (NACI) Statement: Recommendations on the use of pneumococcal vaccines in adults, including Pneu-C-21

**DOI:** 10.14745/ccdr.v51i101112a01

**Published:** 2025-12-12

**Authors:** Eva Wong, Oliver Baclic, Marina I Salvadori, Kyla Hildebrand

**Affiliations:** 1 Centre for Immunization and Respiratory Infectious Diseases, Public Health Agency of Canada, Ottawa, ON; 2 Department of Pediatrics, McGill University, Montréal, QC; 3 Division of Immunology, Department of Pediatrics, University of British Columbia, Vancouver, BC

**Keywords:** National Advisory Committee on Immunization, pneumococcal disease, conjugate pneumococcal vaccine, Canada, adult immunization program, vaccine guidance

## Abstract

**Background:**

Pneumococcal disease in adults includes invasive pneumococcal disease (IPD), an acute and serious communicable disease with manifestations such as meningitis, bacteremia and bacteremic pneumococcal pneumonia. There are more than 100 different serotypes, and the extent of protection provided by a pneumococcal vaccine depends on the vaccine formulation. In July 2024, Health Canada authorized a 21-valent pneumococcal conjugate vaccine (Pneu-C-21), which followed the recent introduction of a 20-valent vaccine (Pneu-C-20) authorized in 2022.

**Methods:**

The National Advisory Committee on Immunization (NACI) reviewed evidence on the epidemiology of IPD in Canada, immunogenicity and safety of Pneu-C-21, and the cost-effectiveness of different pneumococcal vaccines in adult immunization programs. NACI has also considered additional factors, including ethics, equity, feasibility, and acceptability (EEFA).

**Results:**

Differences in the distribution of serotypes causing IPD have been observed before and after the COVID-19 pandemic. The Pneu-C-21 demonstrated comparable immunogenicity to Pneu-C-20 for shared serotypes and higher responses for unique serotypes. The safety profiles of both vaccines are expected to be similar to other pneumococcal vaccines, and the cost-effectiveness of Pneu-C-21 and Pneu-C-20 will depend on regional serotype distribution. The overall impact of Pneu-C-21 compared to Pneu-C-20 is uncertain, but likely to vary over time with age, risk factors, and geography.

**Conclusion:**

NACI now recommends including at least one of Pneu-C-20 or Pneu-C-21 in adult pneumococcal immunization programs. One dose should be given to adults 65 years and older and those 18 to under 65 years at increased IPD risk, regardless of previous pneumococcal vaccination history.

## Introduction

Invasive pneumococcal disease (IPD) in adults represents a significant global public health concern. The burden of disease is predominately attributable to a small number of serotypes of the *Streptococcus pneumoniae* bacteria. The landscape of pneumococcal vaccination in Canada has evolved with the recent authorization of higher-valency pneumococcal conjugate vaccines (Pneu-C), including Pneu-C-15 (Vaxneuvance®, Merck Canada), Pneu-C-20 (PREVNAR 20®, Pfizer Canada), and most recently, Pneu-C-21 (CAPVAXIVE™, Merck Canada).

Currently, all Canadian jurisdictions have publicly funded adult pneumococcal vaccination programs. While specific eligibility varies, the programs generally include adults 65 years of age and older and certain adults 18 to 64 years of age who are at increased risk for IPD. The current national coverage goal for adult pneumococcal vaccination is 80% of adults aged 65 and older receiving one dose by 2025 ((1)). However, this target remains unmet based on coverage estimates.

In 2023, the National Advisory Committee on Immunization (NACI) provided updated guidance recommending Pneu-C-20 for adult programs, with Pneu-C-15 and the 23-valent pneumococcal polysaccharide vaccine (Pneu-P-23) in series as an alternative. NACI has since reviewed additional evidence and provided guidance on the use of pneumococcal vaccines in adults, including Pneu-C-21 ((2)).

## Methods

NACI used a comprehensive, systematic approach for recommendation development, including an analysis of the IPD burden in Canada, and a systematic literature review of the efficacy, effectiveness, immunogenicity, and safety of Pneu-C-21. This included evidence from key clinical trials, published studies, and supplementary data from manufacturers. Evidence was evaluated using the Grading of Recommendations Assessment, Development and Evaluation (GRADE) methodology for studies comparing Pneu-C-21 and Pneu-C-20.

To inform policy recommendations in Canada, economic considerations were assessed through systematic reviews and a *de novo* model-based cost-utility analysis of higher valency pneumococcal conjugate vaccines in adults.

NACI used a published, peer-reviewed framework and evidence informed methodology to ensure that issues related to ethics, equity, feasibility and acceptability (EEFA) were systematically assessed and integrated into the guidance ((3)). The evidence and programmatic considerations were organized by the NACI Secretariat using a process informed by the GRADE framework, and all of the information was used to facilitate development of NACI guidance. Further information on NACI’s evidence-based methods is available in *Evidence-based recommendations for immunization – Methods of the National Advisory Committee on Immunization* ((4)).

## Results

### Epidemiology of invasive pneumococcal disease in Canada

Between 2019 and 2023, Canada’s National Microbiology Laboratory characterized 14,563 isolates of *S. pneumoniae* causing invasive disease in adults, with 39% identified from adults 65 years and older. The majority of IPD cases were caused by vaccine-contained serotypes, with serotypes 3, 4, 12F and 22F being the most common in adults overall during this period.

Age-specific analyses revealed varying serotype distributions. In adults 18 to 49 years, serotypes 4 and 12F were most common, while serotypes 3 and 4 were most common in the 50 to 64 age group. Among those 65 years and older, serotypes 3 and 22F were most frequently isolated. Notably, the proportion of cases caused by Pneu-C-21 serotypes remained relatively stable in adults 65 years and older between 2020 and 2023, while decreasing in younger age groups. There was an overall increasing trend observed in the proportion of Pneu-C-20/non-Pneu-C-21 serotypes across age groups, which was most prominent in the younger adults and less pronounced in adults 65 years and older.

In addition to age-based differences, trends in serotype distribution have also been observed to differ by geography (i.e., Northern Canada vs. the rest of Canada) and by other risk factors for IPD (e.g., individuals who are unhoused).

Additional details are available in the annual report for invasive pneumococcal disease in Canada ((5)).

### Clinical evidence

Clinical trials involving over 7,600 adults demonstrated the immunogenicity and safety of Pneu-C-21 ((6)). In the pivotal double-blind clinical trial, adults aged 18 years and older received either Pneu-C-20 or Pneu-C-21 ((7)). The Pneu-C-21 elicited comparable immune responses to Pneu-C-20 for shared serotypes and higher responses for unique serotypes across all age subgroups.

Generally, higher immune responses were observed in participants aged 18 to 49 years, compared to those 65 years and older. Similar immunogenicity was observed between study participants with and without risk factors for IPD. In vaccine-experienced adults, immune responses were generally comparable between intervention groups, though those previously receiving Pneu-P-23 showed somewhat lower responses.

Safety data from an integrated analysis of multiple trials, involving over 6,000 individuals, showed Pneu-C-21 was well-tolerated. The most common adverse reactions in younger adults (18–49 years) included injection site pain (75%), fatigue (35%), and headache (30%). In adults 50 years and older, adverse events were less frequent, with injection site pain (40%), fatigue (20%), and headache (10%) being most common. The majority of reactions were mild and short-lived (≤3 days), with severe events occurring in less than 1% of participants. The available evidence suggests that there are no meaningful differences in severe adverse events in individuals who received Pneu-C-21 compared to individuals who received Pneu-C-15, Pneu-C-20, or Pneu-P-23.

### Economic considerations

Two systematic reviews were conducted to identify economic evidence for the use of pneumococcal conjugate vaccines in adult populations ((8,9)). The first review, which did not include studies on Pneu-C-21, found that conjugate vaccines (Pneu-C-13 alone or in combination with Pneu-P-23 or Pneu-C-20) may be cost-effective compared to no vaccination for adults aged less than 65 years at higher risk of IPD, at a threshold of $50,000 per quality-adjusted life year (QALY) gained ((8)). A second review identified five economic evaluations assessing the cost-effectiveness of Pneu-C-21 in adults and found that Pneu-C-21 is likely cost-effective in adults within specific age and risk groups; however, the applicability of these evaluations to the Canadian context is limited, as findings are sensitive to the region- and age-specific serotype distribution of pneumococcal disease cases and vaccine price ((9)).

A *de novo* model-based cost-utility analysis ((10)) was conducted by updating a model previously used to assess the cost-effectiveness of Pneu-C-15 and Pneu-C-20 in Canadian adults ((11)). The use of one of the higher valency conjugate vaccines is expected to be a cost-effective strategy (using a $50,000 per QALY threshold) in all population groups considered, with the choice of vaccine being highly dependent on serotype distribution in the target population. These results held even with conservative assumptions about indirect effects and serotype replacement.

### Ethics, equity, feasibility and acceptability considerations

The COVID-19 pandemic has broadly impacted the uptake of routine vaccines, particularly among children. Because paediatric pneumococcal immunization offers indirect protection to adults, it is uncertain how IPD serotype epidemiology will evolve with improved paediatric vaccination, along with the use of higher valency pneumococcal vaccines in both children and adults. While regional epidemiology can better inform the choice of product(s) to use in adult programs, jurisdictions do not necessarily collect and/or have equal access to detailed data, and there may be variability in how or when updated pneumococcal vaccine recommendations are adopted. It is anticipated that program- and individual-level acceptability of the high valency conjugate vaccines will depend on the complexity of the recommendations, and will need to be clearly communicated if programs are recommended to use multiple products, given that Pneu-C-15 and Pneu-C-20 have been recently recommended for use in paediatric and adult immunization programs.

## Discussion

NACI recommendations on adult pneumococcal vaccines for public health program-level decision-making

The following are recommendations for provinces/territories making decisions for publicly funded immunization programs:

• NACI recommends that adult pneumococcal immunization programs should include at least one of Pneu-C-20 or Pneu-C-21 ***(Strong NACI recommendation)***

• NACI recommends that one dose of either Pneu-C-20 or Pneu-C-21 should be given to adults 65 years of age and older, and adults under 65 years at increased risk of IPD (**List 1**), regardless of pneumococcal vaccination history ***(Strong NACI recommendation)***

• For hematopoietic stem cell transplant recipients, both Pneu-C-20 and Pneu-C-21 should be offered after consultation with a transplant specialist. A primary series of three doses should be administered starting 3–9 months post-transplant at four-week intervals, followed by a booster dose at 12–18 months post-transplant ***(Strong NACI recommendation)***

**List 1: Adult risk factors for invasive pneumococcal disease**

**Table:** 

**Medical conditions:** **• Hematopoietic stem cell transplant recipients** **• Chronic cerebrospinal fluid (CSF) leak** **• Cochlear implants, including those who are to receive implants** **• Congenital immunodeficiencies involving any part of the immune system, including B-lymphocyte (humoral) immunity, T-lymphocyte (cell) mediated immunity, complement system (properdin, or factor D deficiencies), or phagocytic functions** **• Immunocompromising conditions or immunosuppressive therapy within the past two years, including use of long-term corticosteroids, chemotherapy, radiation therapy, and immunosuppressive biologics** **• Active malignant neoplasms^a^, including leukemia and lymphoma** **• Candidates and recipients of solid organ or islet transplants** **• Chronic kidney disease, particularly those individuals with stage 4 and 5 chronic kidney disease, and those with nephrotic syndrome, on dialysis or renal transplant recipients** **• Chronic liver disease, including cirrhosis, biliary atresia and chronic hepatitis** **• HIV infection** **• Functional or anatomic asplenia (congenital or acquired) or splenic dysfunction, including sickle cell disease and other hemoglobinopathies** **• Chronic neurologic conditions that may impair clearance of oral secretions** **• Chronic heart disease requiring regular medication and follow up for ischemic heart disease, congenital heart disease, chronic heart failure, or hypertension with cardiac complications** **• Diabetes mellitus, particularly in those over 50 years of age** **• Chronic lung disease (particularly chronic obstructive pulmonary disease, emphysema, bronchiectasis, interstitial lung fibrosis, and cystic fibrosis), including asthma that required medical care in the preceding 12 months** **Social, behavioural, and environmental factors:** **• Individuals who are unhoused** **• Individuals living in communities or settings experiencing sustained high IPD rates, including those who are in residential care^b,c^** **• Smoking, particularly in those over 50 years of age** **• Substance use (i.e., alcohol misuse, cocaine use, and injection drug use)** **• Occupational risk with long-term continuous exposure to metal fumes (i.e., welders)**

Abbreviation: IPD, invasive pneumococcal disease^a^ Immune compromised status can vary over time depending upon the disease state, which may or may not involve immunosuppressive medication^b^ Including long-term care homes^c^ Individuals should be vaccinated with a vaccine that covers serotypes circulating in the communityNote: It should be noted that some conditions listed above carry higher risk than others. In adults, risk increases at a population level with advancing age (beginning at age 50). In addition, having multiple risk factors at once may also increase risk for an individual. Program-level recommendations may focus on the populations particularly at risk

### Additional guidance on pneumococcal vaccines

• The aim of the pneumococcal vaccine program is to protect adults at high risk for infection and medical complications. With the uncertainty around ongoing IPD epidemiology and as vaccine effectiveness data accrues, simplicity and flexibility in the recommendations are needed to allow jurisdictions the ability to consider how to best achieve the highest possible benefit in the targeted populations.

• The choice of the vaccine(s) used will depend on determining the most suitable vaccine(s) based on regional epidemiology, vaccine eligibility (including prior immunization with a pneumococcal vaccine), and programmatic considerations.

• For adults who have previously received pneumococcal vaccination, the choice of vaccine and interval from the last dose will depend on the type of vaccine previously received and time since vaccination. Based on expert opinion, the recommended interval between Pneu-P-23 and either Pneu-C-20 or Pneu-C-21 is now one year; however, an interval as short as eight weeks may be considered in those who might be anticipating initiation of immunosuppressive treatments or who have diseases that might lead to immunodeficiency.

• At the individual level, it should also be noted that adults 18 years of age and older who are not included for higher valency pneumococcal vaccine in publicly funded programs may opt to receive higher valency pneumococcal vaccination in consultation with their healthcare providers.

## Conclusion

NACI now recommends that adult pneumococcal vaccination programs use at least one of Pneu-C-20 or Pneu-C-21. The impact of the recent changes to Canadian pneumococcal vaccine programs may not be evident in the short-term, as it can take several years to observe notable changes to IPD serotype epidemiology. NACI will continue to monitor the epidemiology of pneumococcal disease, including IPD, and will update the recommendations on the use of pneumococcal vaccines as needed.
